# Trafficking of ThermoTRP Channels

**DOI:** 10.3390/membranes4030525

**Published:** 2014-08-19

**Authors:** Clotilde Ferrandiz-Huertas, Sakthikumar Mathivanan, Christoph Jakob Wolf, Isabel Devesa, Antonio Ferrer-Montiel

**Affiliations:** 1 Instituto de Biología Molecular y Celular, Universidad Miguel Hernández, Alicante 03202, Spain; E-Mails: clotilde.ferrandiz@umh.es (C.F.-H.); smathivanan@umh.es (S.M.); cwolf@umh.es (C.J.W.); 2 BIOFISIKA, the Basque Center for Biophysics, UPV/EHU-CSIC-FBB, Bilbao 48940, Spain

**Keywords:** TRP, thermoTRP, exocytosis, SNARE, protein-protein

## Abstract

ThermoTRP channels (thermoTRPs) define a subfamily of the transient receptor potential (TRP) channels that are activated by changes in the environmental temperature, from noxious cold to injurious heat. Acting as integrators of several stimuli and signalling pathways, dysfunction of these channels contributes to several pathological states. The surface expression of thermoTRPs is controlled by both, the constitutive and regulated vesicular trafficking. Modulation of receptor surface density during pathological processes is nowadays considered as an interesting therapeutic approach for management of diseases, such as chronic pain, in which an increased trafficking is associated with the pathological state. This review will focus on the recent advances trafficking of the thermoTRP channels, TRPV1, TRPV2, TRPV4, TRPM3, TRPM8 and TRPA1, into/from the plasma membrane. Particularly, regulated membrane insertion of thermoTRPs channels contributes to a fine tuning of final channel activity, and indeed, it has resulted in the development of novel therapeutic approaches with successful clinical results such as disruption of SNARE-dependent exocytosis by botulinum toxin or botulinomimetic peptides.

## 1. Introduction

Transient receptor potential (TRP) channels consist of a large family of ion channels that play a wide diversity of physiological functions [1,2,3]. Expressed in a large number of tissues from nerve to epithelial cells, genetic studies have linked mutations in these ion channels to human diseases [3,4]. The majority of TRP channels are non-selective cation channels that permeate Na^+^ and K^+^, and most of them with significant Ca^2+^ selectivity. In mammals, this family consists of 28 different TRP members grouped in 7 subfamilies: TRPC (classical or canonical), TRPV (vanilloid), TRPM (melastatin), TRPA (ankyrin-like), TRPPP (polycysteine), TRPML (mucolipin) and the TRPN (no mechanoreceptor potential C (NOMPC) [1,2,3], while the TRPY is present in yeast.

**Figure 1:**
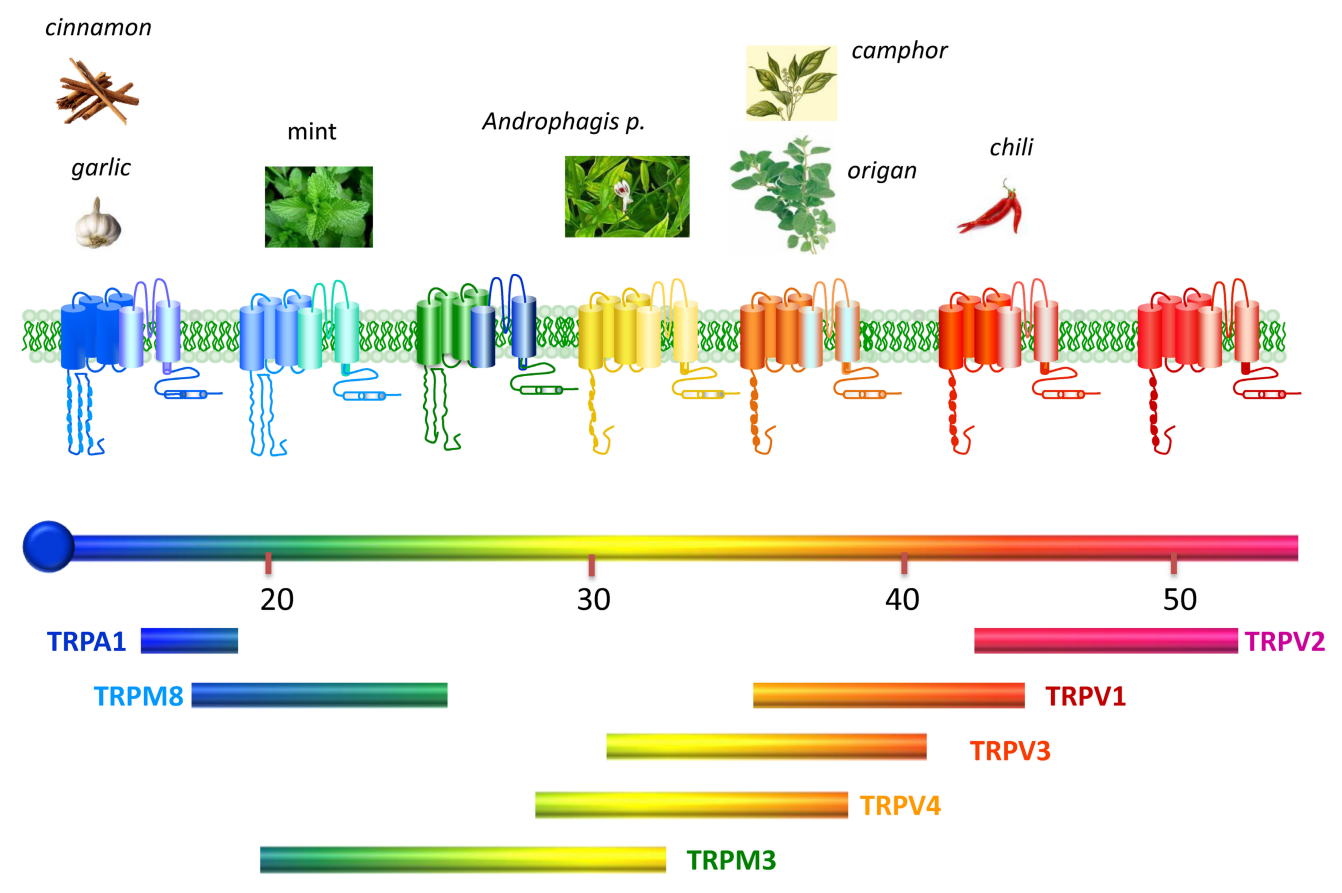
Thermotransient receptor potential (TRP) channels. Structurally thermoTRP are tetramers and each subunit contains six transmembrane domains (S1–S6), a hydrophobic pore loop linking transmembrane S5 and S6, and large cytoplasmic *N*- and *C*-terminals (NB not drawn to scale). All thermoTRPs have a variable number of ankyrin repeat domains in the *N*-terminus, except TRPM8 which has none and instead contains TRPM homology region. ThermoTRPs display distinct thermal thresholds from very noxious cold (TRPA1) to harmful hot (TRPV2). Each thermoTRP is also activated by specific natural or synthetic compounds, known to induce the relevant thermal and pain sensations in humans. Adapted from [10].

Thermosensory channels, also named “thermoTRPs”, define a subfamily of the TRP channels that are activated by changes in the environmental temperature, from noxious cold (<15 °C) to injurious heat (>42 °C) (Figure 1). Acting as integrators of several stimuli and signalling pathways, dysfunction of these channels, for instance, contributes to thermal hyperalgesia and allodynia under pathological painful conditions such as inflammation or cancer [4,5,6,7]. For this reason, thermoTRPs have become pivotal drug targets, and the development of therapeutic compounds for pharmacological intervention is actively pursued by the academy and the industry [8,9].

The cellular activity of polymodal thermoTRP channels is subjected to a complex modulation encompassing from posttraductional modification, usually by phosphorylation/dephosphorylation, to regulation of their membrane expression [11]. Notably, recruitment of thermoTRP channels to the cell surface has been revealed as a pivotal mechanism for algesic sensitization of sensory neurons [12]. Furthermore, yeast-screen assays have identified a large number of proteins implicated in vesicular trafficking as partners of thermoTRP channels [13]. Accordingly, the cellular and molecular mechanisms involved in the transport and mobilization of these channels to the cell membrane are currently being an area of intense investigation.

Cumulative evidence suggest that thermoTRP channels are actively transported by both constitutive and regulated pathways depending on the cell type and cellular environment. Here, we will cover the recent advances in the membrane trafficking of the classical thermosensory channels, namely TRPV1, TRPV2, TRPV4, TRPM8, TRPM3 and TRPA1. It should be mention that TRPV3 is also one of the thermoTRPs; however, we will not discuss this channel since until now there are no data describing any finding on its trafficking. This review will focus on the progress carried out on this fascinating topic and will highlight the tenet that regulated membrane insertion of thermoTRPs channels contribute to a fine tuning of channel activity. The clear contribution of this mechanism to the final channel function has opened exciting research lines driven to the development of novel therapeutic approaches with successful clinical results in the treatment of several painful pathologies.

## 2. ThermoTRP Channels

ThermoTRPs are expressed as homotetramers, although heterotetramers have been also proposed for some of them [3]. All of them present six transmembrane domains (S1–S6), with a pore region between S5 and S6, and with N- and C-terminal cytosolic domain. The N-terminus has several ankyrin repeats or TRPM homology regions, while the C-terminus contains motives for multimerization, protein consensus sequences for kinases, and the transient receptor potential (TRP) domain important for channel gating.

### 2.1. TRPV1

Transient receptor potential vanilloid 1 (TRPV1) is the archetypal and the most studied thermoTRP channel. TRPV1 is a 95 kD protein with 838 aa [14] whose putative structure has been recently proposed by cryo-electron microscopy [15,16,17]. Functionally TRPV1 exists as homotetramer [16] and there are at least six ankyrin repeats in the N-terminus [18,19] essential for channel function and numerous protein-protein interactions [20,21]. The C-terminal contains subdomains involved in different channel functions: (i) determinants for subunit tetramerization [22]; (ii) the TRP domain [23,24]; and (iii) consensus sequences for phosphoinositides [25] and protein kinases [26].

This polymodal channel can be activated by noxious heat (>42 °C), acidic pH (pH < 5.9), voltage and numerous chemical ligands [27,28]. Capsaicin is the main known natural agonist of TRPV1, but it can be also activated by others pungent extract, toxins or even some endogenous ligands such as anandamide (known as endo-vanilloid) [29,30]. TRPV1 shows equal selectivity for Na^+^, K^+^, Li^+^, Cs^+^ and Rb^+^, but moderate for divalent cations such as Ca^2+^ and Mg^2+^ [14,31,32].

TRPV1 is subjected to complex regulation, from gene expression to post-translational modification as well as subcellular compartmentalisation and association with regulatory proteins [33,34]. The activity of TRPV1 can be rapidly regulated by phosphorylation of several key residues leading to an increased sensitivity to both chemical and thermal stimuli. Kinases such as protein kinase C (PKC), protein kinase A (PKA), calmodulin-dependent kinase (CaM) and Src-kinase increase TRPV1 activity, while calcineurin-mediated dephosophorylation at the same sites produces TRPV1 desentitization [11]. As consequence, the formation of signalplexes or the physical assembly of signalling molecules into discrete macromolecular entities is an essential pathway that influences TRPV1 activity [11].

This thermoTRP channel is widely distributed in neuronal as well as non-neuronal tissues. In the peripheral nervous system, TRPV1 is highly expressed in dorsal root (DRG), trigeminal (TG) and nodose (ND) ganglia [14]. Mainly present in small and medium peptidergic neurons, and to a lesser extent in non-peptidergic nociceptors [29,35]. Strongly localized on lamina I and outer portion of II in the dorsal horn [36,37,38], the presence of TRPV1 in sensory nerves and in areas involved in detection, transmission and regulation of pain reveals its key role in nociception and neurogenic inflammation [39]. In the central nervous system, although with some initial controversy on its brain distribution [40,41,42], the role of TRPV1 in synaptic transmission, neurotransmitter release and plasticity is already described by several groups [43,44], while a potential contribution in cognition, perception and neuropsychiatric disorders is being proposed [45].

There is an extensive list of non-neuronal tissues expressing TRPV1 channel [46,47]. For instance, TRPV1 participates in many aspects of skin biology [48,49], joint homeostasis and pathophysiology [50,51,52], produces vasodilatation and extravasation through the release of neuropeptides from the innervating sensory neurons [53,54], but it can directly evoke constriction [55]. Other physiological mechanisms can be modulated by TRPV1 such as cough [56] or to control bladder function and overactivity [57].

Nevertheless, the main known role of TRPV1 is as thermosensor transducing physical, chemical and thermal noxius stimuli. With a prominent role during inflammation, TRPV1 is responsible for thermal development and maintenance. In fact, both pharmacological and knockout studies have demonstrated its pivotal role on pain. TRPV1 is overexpressed in several chronic painful pathologies [58] such as rheumatoid arthritis [59,60], osteoarthritis [61], bone cancer-induced pain [62] and several neuropathies [63,64] among others. All these evidences promoted TRPV1 as an interesting pharmacological target to develop new analgesic treatments especially in diseases with an inflammatory component. In this regard, clinical trials have already shown a hyperthermic response upon treatment with some TRPV1 antagonists [65]. It is now known that TRPV1 is involved in the maintenance of normal body temperature [66], and recent studies propose that antagonists that do not block activation of TRPV1 by acidic solution apparently do not induced hyperthermia [67].

### 2.2. TRPV2

Transient receptor potential vanilloid 2 (TRPV2) is a thermoTRP channel activated by high temperatures (≈52 °C). Cloning of human and rat TRPV2 was made based on their homology to TRPV1, hence it has been also referred to as vanilloid receptor like protein 1 (VRL-1) or osm-9-like TRP channel 2 (OTRPC2). Structurally, TRPV2 consists of 740 aa with six ankyrin repeats domains (ARD) in its large N-terminus. The membrane proximal domain is an important region that connects TRPV2-ARD to the S1 and apparently modulates temperature sensitivity [68]. The C-terminus contains the TRP domain important for oligomerization [23], as well as domains for phosphotidylinositol 4,5- bisphospate (PIP_2_) [69] and CaM binding [70]. The N-glycosylation motif between S5–S6 loop is required for TRPV2 plasma membrane expression, since only the glycolsylated form reaches cell surface while the non-glycosylated remains in the cytosol [71].

TRPV2 is permeable to divalent cations with high selectivity for Ca^2+^ [72]. TRPV2 is strongly activated by 2-aminoethoxydiphenyl borate [73], diphenylyboronic anhydride, and derivatives of *Cannabis sativa* like cannabidiol, similar to other TRP channels such as TRPV1 or TRPA1. Probenecid is a potent activator of TRPV2 [74], while other endogenous activators like lysophosphotidyl choline can activate TRPV2 as well [75].

Similar to TRPV1, TRPV2 function can be rapidly regulated by post-translational modifications such as phosphorylation/dephosphorylation by PKA or PI3-kinase [76,77] or desensitization by Ca^2+^. Though TRPV2 does not contain the binding sites for CaM, ATP, or PIP_2_, a recent novel Ca^2+^-dependent binding site for CaM in the C-terminal fragment has been proposed, but probably CaM binding may not be functionally coupled to TRPV2 desensitization [69].

Expressed in several tissues, TRPV2 shows different physiological functions. In the nervous system, it is highly expressed in sensory neurons, being present in large and medium diameter DRG neurons with higher heat threshold and slow activating currents [78], while in TG is also located in large diameter neurons [79]. The expression of TRPV2 in sensory neurons and its activation by noxius heat [72] suggested a role in nociception. Nevertheless, deletion of TRPV2 gene expression does not affect thermal or mechanical sensing in mice [80]. During peripheral chronic inflammation, TRPV2 expression is increased, [81] and contributes to noxious heat hyperalgesia, especially in the absence of TRPV1. However, since acute nociception and thermal hyperalgesia are not impaired [80], the role of TRPV2 in thermosensing still remains highly controversial. The presence of TRPV2 in spinal cord and in different brain areas reveals other roles for this channel such as axonal outgrowth [82] or modulation of astrocyte function [75].

Outside the nervous system, TRPV2 mediates oxytocin and vasopressin release [83], participates in cardiac contractility and Ca^2+^-regulation [84], acts as an important stretch sensor in myocytes [85] and contributes to osteoclastogenesis [86]. Interestingly, TRPV2 shows an important role in immune response being expressed in several immune cell types [87], and in several cancer processes, like urothelial carcinoma in bladder [88] or glioblastomamultiforme [89], with a relevant role on cell migration [90]. Notably, TRPV2 has been involved in some hereditary diseases, such as muscular dystrophy [91], being a player in the pathogenesis of myocyte degeneration, and cell stretch increases TRPV2 translocation to the sarcolemma leading to external Ca^2+^ overloading in animal models and patients [92].

### 2.3. TRPV4

Transient receptor potential vanilloid 4 (TRPV4), also recognized as TRP12, OTRPC4 or VR-OAC, was initially detected as a channel activated by hypotonicity [93]. All mammalian TRPV4 homologues share high degree of sequence identity (95%–98%) [94]. In the N-terminus, the 6 ankyrin repeated domains are involved in TRPV4 protein-protein interactions [95], and seem to be related to self-association of N-termini into the tetrameric structure [96], acting as molecular determinants of subunit assembly and subsequent processing of the channel [97]. In the N-terminus, TRPV4 contains a proline rich domain involved in mechano-sensitive properties [98]. Like other TRP channels, TRPV4 is subject to dual Ca^2+^-dependent regulation, with channel activity potentiated and inactivated during agonist-dependent activation in the presence of the divalent cation [99]. The C-terminus comprises several putative CaM binding sites basis of the Ca^2+^-dependent potentiation process [100].

Nowadays, TRPV4 is already defined as a polymodal channel activated by various stimuli ranging from physical stimuli to chemical activators, being considered as a mechano- or osmo-sensor and a moderate heat sensor (between 24 and 27 °C) [101]. Agonists of TRPV4 include the endocannabinoid anandamide, the metabolite arachidonic acid [102], bisandrographolide A [103], 4-alpha-phorbol 12,13-didecanoate [104], acetylcholine [105], apigenin [106], or dimethylallyl pyrophosphate [107].

This thermoTRP is a non-selective cationic channel with higher permeability to Ca^2+^ and Mg^2+^ that Na^+^ [108]. Apart from the complex Ca^2+^ dependent regulation of TRPV4 [99], gating of the channel can be modulated by other mechanisms. For instance, PIP_2_ interaction with N-terminus increases channel activation by hypotonicity and heat, while disruption of this interaction prevents channel activation or sensitization through the PLC pathway [109]. Gating of TRPV4 can be also regulated by both PKC-independent and dependent pathways [110,111,112].

TRPV4 shows a wide range expression and physiological functions, and it is involved in the development of several pathological conditions [94]. For instance, TRPV4 regulates cell-cell junction, maintains skin barrier homeostasis playing a role in cutaneous thermoregulation [113,114], is determinant in bone physiology, joint homeostasis, bone remodelling, and chondrocyte differentiation [115,116]. This channel is also present in airway smooth muscle cells [117], different types of renal cells [118], and aortic endothelial cells [119]. In the nervous system, TRPV4 is a key regulator of hippocampal neural excitability [120] and participating in astrocytes homeostasis [121]; however, TRPV4 presence in DRG, TG and ND neurons promoted its study on pain pathophysiology [122]. Thus, TRPV4 contributes to thermal and mechanical hyperalgesia [123,124], several peripheral preclinical and clinical neuropathies [125,126,127] and a preclinical model of migraine [128]. TRPV4 has been also implied in cystic fibrosis [129], human skin cancer [130], post burn pruritus, and liver and renal associated pathologies among other.

Interestingly, TRPV4 has gained medical and clinical interest since mutations in the TRPV4 gene can result in genetic disorders like Brachyolmia, Charco-Marie Tooth type 2C, spinal muscular artrophy and hereditary motor and sensory neuropathy type 2 [131]. The mutation of TRPV4 does not seem to modify directly the channel activity. However, it seems that it is the environment and the interaction of other factors that modulate oligomeraziation, trafficking and degradation of TRPV4, enhancing or reducing the activity of the channels [131].

### 2.4. TRPM3

Transient receptor potential melastatin-3 (TRPM3) is a recently included member of the thermoTRP family that belongs to the melastatin subfamily of TRPs. Intriguingly, TRPM3 is a heat sensitive channel with limited homology to the TRPV members [132]. Human TRPM3 has 57% sequence identity and 67% similarity with human TRPM1, and both genes encode a microRNA [133], therefore often TRPM3 is grouped with TRPM1 [134]. The cytosolic C-terminus contains the TRP-box and the typical coiled-coil domain of melastatin subfamily members, which is important for assembly for other TRPM, but not yet for TRPM3. Important domains for protein-protein interaction such as CaM and PIP_2_ binding domains are present in the N-terminus [135].

Several splice variants of TRPM3 are known which are divided in three groups depending on their first exon. TRPM3α (α1–α5) isoforms start with exon 1 and lack exon 2, while TRPM3β isoforms (β1–β17) start with exon 2. A third group is composed of isoforms starting with exon 3 [136]. This plethora of splice variants together with the microRNA seems to facilitate a fine tuning of the channel expression level. In contrast, alternative splicing in exon 24 leads to two different pores and influences cation selectivity [137].

TRPM3 channels are non-selective cationic channels activated by both physical (heat, voltage and hypotonic cell swelling) and chemical stimuli such as the neuroactive steroid pregnenolone sulfate, d-erythro-sphingosine or internal Ca^2+^ store depletion. However, less is known about the post-translation mechanisms that regulate TRPM3 channel activity. Indeed, there is no evidence of phosphorylation regulation through the well-known PKC and PKA, and there is limited information on the functional implication of calmodulin and PIP_2_ association.

The physiological and/or pathological role of TRPM3 still remains to be deeply elucidated since research on this channel has just started. Nevertheless, TRPM3 has recently drawn much attention since it has been described as a heat sensing channel expressed in nociceptors [132]. Expressed in a large number of small-diameter DRG and TG neurons, TRPM3 detects noxious heat, alike as TRPV1 but with more lower and broad range of temperatures, and is involved in heat-, but not cold-, mediated hyperalgesia [132,138]. Unlike with TRPV1, inhibition or knockout of TRPM3 does not affect the homeostasis of body temperature. Most importantly, TRPM3-deficient mice exhibit deficits in responses to noxious heat and development of inflammatory heat hyperalgesia [132,138].

TRPM3 expression is also found in the brain [139,140], eyes, reproductive system, smooth muscle cells, pituitary gland, adipose tissue, cardio vascular system [2], and pancreas [132,141].

### 2.5. TRPM8

In contrast to the above described thermoTRPs, transient receptor potential melastatin 8 (TRPM8), also known as Trp-p8, is a 1104 aa cold-sensitive ion channel [142]. While the C-terminus appears involved in temperature mediated gating [143] and contains the TRP-domain, the N-terminus harbours the four conserved regions with homology sequences (MHR) characteristic of the TRPM subfamily [144]. Serine residues on the S3–S4 extracellular linker are important for channel gating [145], and there are hints that S4 and the S4–S5 linker are part of the voltage sensor [146]. While the first 39 aa are not crucial for channel activity [144], the disulphide bond between Cys929 and Cys940 is essential [147]. Interestingly, residues 40–86 seem involved in localization of the TRPM8 tetramer in the plasma membrane and *in vivo* stabilization [144,148].

The typically observed temperature threshold for recombinant TRPM8 activation (17–25 °C) [148,149,150] does not account for its function *in vivo* where it responds to skin temperatures [151]. The original threshold is only recovered in primary sensory neurons but not in other neurons, that suggests a regulation via endogenous factors expressed solely in these neurons [152]. Activation of TRPM8 leads to a negative shift in the voltage-dependent activation that facilitates channel opening at negative potentials [153,154,155]. Apart from cold temperature, a broad range of natural and synthetic compounds activate TRPM8: menthol and derivatives, eucalyptol, icilin, hydroxycitronellal, heliotropyl acetone, helional, geraniol, and linalool [153,156,157].

TRPM8 can be regulated by various intracellular secondary messengers and signalling pathways that allow a fine tuning of channel activity by the cellular environment [158]. A well-known regulator of TRPM8 activity is PIP_2_, which directly interacts with the TRP domain and modulates cold, menthol or voltage activation [159,160]. Ca^2+^-dependent CaM provides a rapid desensitization mechanism of TRPM8 [161]. Besides, Ca^2+^ influx through TRPM8 activates PLC isoform which hidrolyzes PIP_2_ and decreases channel activity, being responsible of Ca^2+^-dependent tachyphylaxia [159,162,163]. Therefore, activation of any receptor that activates PLC and promotes PIP_2_ depletion may cause both inactivation and the desensitization of TRPM8, such as phorbol ester, bradykinin, or nerve growth factor (NGF) [164,165,166]. Additionally, although yet controversial, activation of GPCR/Gi followed by adenylate cyclase (AC)/cAMP/PKA or cPLA_2_ phopholipase2-arachidonic acid pathway results in a decrease in channel function [167]. On the contrary, positive regulation of TRPM8 channel activity can be achieved through Gs/AC/cAMP/PKA signalling pathway [168] or by the main products of iPLA_2_ [169]. In this regard, it should be mentioned that basal activity of TRPM8 depends on phosphorylation by PKA on residues Ser9 and Thr17 [168]. Other inhibitors of TRPM8 function include GCPR/Gαq, endovanilloid anandamide and several cannabinoids [170].

TRPM8-expressing fibres innervate tissues such as skin, mucosa or visceral organs like the colon [171,172]. For a long time, cooling or topical application of menthol has been used for their analgesic effects. This evidence, together with TRPM8 presence in DRG and TG neurons [173,174], suggests a potential role of TRPM8 in thermal sensing and nociception. In contrast to the pro-algesic activity of other ThermoTRPs, TRPM8 mediates analgesia in neuropathic and inflammatory pain models [175,176,177,178]. TRPM8 is a major integrator of cold and menthol sensitivity and allodynia, and it is required for behavioral responses to innocuous cool, noxious cold, injury-evoked cold hypersensitivity, cooling-mediated analgesia, and thermoregulation [179,180].

Other tissues expressing TRPM8 are skeletal and smooth muscle, epithelia of the prostate, lungs, bladder and urogenital tract [181,182], although the role of TRPM8 in these tissues is still not understood. Recent observations of TRPM8 expression in vagal neurons innervating bronchopulmonary tissue have brought up TRPM8 as a drug target for various respiratory disorders [183]. Because up-regulation of TRPM8 expression has been observed in various types of cancer including prostate, breast, lung and colon [184], this cold thermorTRP has been proposed as a marker for cancer tracing [185]. Other diseases where TRPM8 is involved include dry-eye-syndrome [186,187], amyloidotic polyneuropathy [188], and diseases of the urogenital tract like overactive bladder syndrome and pain bladder syndrome [177,181].

### 2.6. TRPA1

Transient Receptor Potential Ankyrin 1 (TPRA1) is the only member of the ankyrin subfamily found in mammals. Originally called ANKTM1, TRPA1 was identified by a homology search for ankyrin domains [189]. Its structure is distinct from other TRP channels as it is the only member with an extended ankyrin repeat domain in the N-terminus [190]. The N-terminus (half the size of the protein) contains between 14 and 18 ankyrin repeats that probably are important for protein-protein interactions and insertion of the channel into the plasma membrane [96,191]. Because of the unusual large N-terminal ankyrin repeat domain, it is also possible that TRPA1 is involved in mechanosensation, in which the N-terminal could act as a link between mechanical stimuli and channel gating [96]. The N-terminal region contains a large number of cysteines, some of which can form a complex network of protein disulphide bridges within and between monomers [96,192,193], targets for electrophilic TRPA1 activators, but cysteines outside the N-terminal region may also contribute to channel gating [194,195]. The N- and C-termini have been suggested to contain binding sites for Ca^2+^ that can both sensitize and desensitize TRPA1 [2,196,197,198,199,200].

TRPA1 is a non-selective cation channel permeable to Ca^2+^, Na^+^ and K^+^. TRPA1 was initially suggested to function as a detector of noxious cold, less than 17 °C [3,201], and to account for a component of cold sensitivity not mediated by TRPM8 [189]. Although this hypothesis remains controversial, current evidence suggest that TRPA1 plays, little, if any, role in acute cold sensation but more likely contributes to injury-evoked cold hypersensitivity [202,203,204,205]. Irrespective of this cold controversy, there is a widespread agreement that TRPA1 plays an important role in chemonociception by serving as a detector for chemical irritants that elicit acute and inflammatory pain [206,207,208]. TRPA1 is activated by a diverse assortment of pungent or irritating reactive chemical compounds including those found in mustard oil (MO, allyl isothiocyanate), cinnamon oil (cinnamaldehide), gas exhaust (acrolein), raw garlic and onions (allicin) and formalin (formaldehyde) [189,197,209,210,211,212] or endogenous compounds such as H_2_O_2_, the alkenyl aldehydes 4-hydroxynonena, 4-oxo-nonenal, 4 hydroxyhexenal and the cyclopentenone prostaglandin, 15-deoxy-δ-(12,14)-prostaglandin J(2) 15d-PGJ_2_ [213,214]. All of them activate the channel by covalent modification of cysteines and lysines in the N-terminus eliciting a painful burning or prickling sensation [215,216].

TRPA1 can be directly activated by Ca^2+^ which exerts dual effects on the channel, including initial activation or potentiation, followed by long-lasting inactivation [197,217]. It has been suggested that Ca^2+^ exerts its effects on TRPA1 by interacting directly with an intracellular domain(s) of the channel [200,218]; however, the underlying mechanism remains controversial [191,200]. In addition to activation by PLC-evoked release of Ca^2+^ from intracellular stores, TRPA1 may also amplify responses initiated by other Ca^2+^-permeable channels, such as TRPV1, further promoting sensitization to thermal or chemical stimuli [208,211].

Pro-inflammatory and pain producing agents, such as bradykinin, histamine, prostaglandins, and trypsin, acting on GPCRs stimulate via PLC or AC, directly or indirectly activate TRPA1 [219]. The downstream products of PLC-activation do not contribute to sensitization of TRPA1. Thus, the remaining possible mechanism is the consequence of membrane PIP_2_ hydrolysis by PLC activation. A similar mechanism was also found in B2R-induced potentiation of TRPA1 [217]. Although, recent pharmacological studies further suggest that some GPCRs, such as the chloroquine activated itch receptor MrgprA3, stimulates TRPA1 through a mechanism involving direct coupling to Gβγ subunits [220].

In sensory neurons from TG, DRG and ND, TRPA1 is expressed in both peptidergic and non-peptidergic neurons [206,221,222]. TRPA1 is mostly found in a subpopulation of TRPV1-positive neurons, but non-TRPV1-containing neurons expressing TRPA1 exist. TRPA1-positive C-fibres densely innervate the skin, airways and gastrointestinal tract. This location, along with the robust activation of TRPA1 by inflammatory mediators and the ability of the channel to promote inflammation, have implicated TRPA1 in pain perception, sensory hyperreactivity, or disease progression of arthritis, asthma, dermatitis, inflammatory bowel disease, and pancreatitis. Finally, TRPA1 has been also related in thermal and mechanical allodynia caused by many neurotoxic cancer chemotherapies together with TRPM8 and TRPV1 [2,211]. Outside sensory neurons, TRPA1 is found in epithelial cells, melanocytes, mast cells, fibroblasts, odontoblasts, and enterochromaffin cells and β-cells of the Langerhans islets. Notably, many of these cells have sensory properties and crosstalk with nearby nociceptors [2].

## 3. Trafficking of ThermoTRP

**Figure 2:**
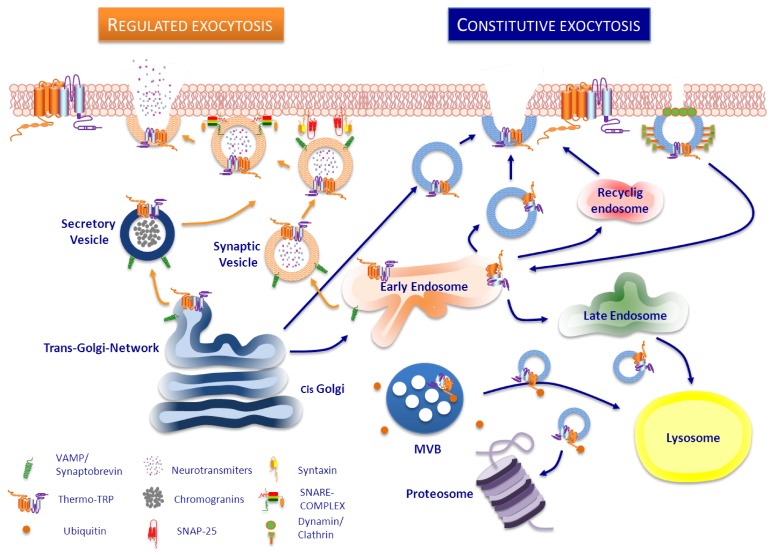
Trafficking pathways of ThermoTRP channels to the plasma membrane. A neuron is illustrated here as example. (**Left** side). Regulated vesicles such as synaptic vesicles, filled with neurotransmitters, or secretory vesicles such as large dense core vesicles, that contain neuropeptides, can also store thermoTRP channels which are delivered to the cell surface upon stimulation by secretagogues. Docking, priming and fusion of these vesicles into the plasma membrane is a SNARE-complex and Ca^2+^-dependent process. (**Right** side). In the constitutive pathway, thermoTRP channels reach plasma membrane by a constant exocytosis from the trans-Golgi directly or via early endosomes (EE). Membrane levels of thermoTRP channels can be also regulated through the classic clathrin-dependent endocytosis pathway, in which dynamin form a ring around the neck of clathrin-coated pit, leading to formation of clathrin-coated vesicles which transport membrane proteins to EE. Once there, proteins could be sorted either into late endosomes and lysosomes or recycled to cell surface via recycling endosomes. ThermoTRP channels destined to degradation could be sorted into intraluminal vesicles of a multi vesicular body (MVB) and lead to lysosome or could be ubiquitinated and lead to proteasome.

The function of any membrane protein can be up- or down-regulated by altering the number expressed at the cell surface. This type of regulation can involve synthesis of new protein or a change in the rate of degradation. However, a rapid change can be achieved by insertion into and retrieval from the surface of ready-synthesized molecules stored in intracellular compartments located beneath plasma membrane [223]. In general, like in all membrane proteins, surface expression of thermoTRP can be regulated in several manners with the final aim of keeping channel homeostasis (Figure 2). New channels are recruited to the cell surface when required, and the pre-existing channels are recycled by endocytosis/exocytosis, regulated by ubiquitination, internalized to proteasomal degradation [224] or degradated by lysosome pathway from multivesicular bodies [225].

There are two mechanisms involved in the exocytotic trafficking of any membrane receptor to the plasma membrane: the constitutive and the regulated exocytosis pathways. After folding, membrane proteins are assembled in the ER and Golgi cisternae, then sorted into vesicles and finally trafficked to the membrane. The constitutive route keeps membrane homeostasis and turnover through a fine tuning of delivery and retrieval of membrane components. This pathway does not respond to signals but can be regulated by a cascade of protein-protein interactions. On the contrary, regulated exocytosis is produced in response to stimuli that provokes mobilization, docking and fusion of intracellular pool of vesicles located near the plasma membrane. This pathway is commonly used in secretory and excitable cells for release of signalling molecules, and excellent reviews have been published on this topic [223,226,227,228,229,230].

Molecular mechanism involved in both types of exocytosis involved a cascade of protein-protein interactions [223,230]. Accordingly, it is important to better understand which interactions are present and how they affect channel expression and function. ThermoTRPs interact with a large number of signalling molecules, scaffolding and trafficking proteins [2,3,13]. Some of these interactions has been already shown essential for correct sorting, transport and delivery of the receptors to the membrane and for their functionality.

### 3.1. TRPV1

With the raising interest of TRPV1 as therapeutic target, this channel was the first, and without any doubt, the most studied thermoTRP. Although, clinical studies are already ongoing on this target, there is still limited and fragmented available information on TRPV1 trafficking. Despite the increasing number of potential TRPV1 interaction partners identified using yeast two-hybrid screening, proteomic screening or immunoprecipitation followed mass spectrometry, the mechanisms and regulatory pathways that control TRPV1 traffic remain to be further elucidated. Among all these interactions, better knowledge of those interactions involved in TRPV1 trafficking could open new potential therapeutic approaches [2,3,13,231].

Ion channels such as TRPV1 are usually key components of macromolecular assemblies that act as functional protein networks [11]. One important component of the TRPV1 signal complex is the γ-amino butyric acid A-Type (GABA_A_) receptor associated protein (GABARAP). Associated with TRPV1 in HEK293 and rat DRGs, GABARAP enhances TRPV1 channel expression, traffic and clustering on the plasma membrane. In addition, it modulates TRPV1 functional activity at the level of channel gating and desensitization [232]. GABARAP slightly augments TRPV1 internalized fraction without affecting the kinetics decay, indicating that this anchor protein increases TRPV1 expression and regulates the receptor cycling between the plasma and the intracellular compartments without altering the receptor degradation rate. Additionally, the presence of GABARAP selectively increased the interaction of tubulin with the C-terminal domain of TRPV1, and both proteins are known to bind independently to microtubules [233,234]. Functionally, TRPV1 interaction with tubulin is increased drastically by GABARAP, which promotes and stabilizes the formation of the complex. Microtubule disruption and GABARAP had an attenuating effect on TRPV1 currents activated by capsaicin, and on the rate of desensitization. Thus, it seems that microtubule dynamics is important regulating these signalling complexes.

TRPV1 interacts physically with tubulin dimer as well as with polymerized microtubules [233], and shows multiple tubulin-binding sites [232,235]. There is a cross-talk between TRPV1 and microtubule cytoskeleton at various levels [236], although the exact mechanism remains still unclear. TRPV1 activation results in rapid disassembly of microtubules [235], while microtubule cytoskeleton helps to form TRPV1 tetramers from dimers at the membrane and preserves the functionality of TRPV1 [237]. Interestingly, upon disruption of microtubule dynamics and disassembly by nocodazole, the interaction with TRPV1 is lost inducing receptor self-aggregation with partial loss of activity, which may be a consequence of an interference with the vesicular trafficking of TRPV1.

Another protein recently described to regulate TRPV1 transport is the cyclin-dependent kinase 5 (Cdk5), which is involved in many cellular processes of the nervous system, including vesicle transport from the Golgi to neurites [238], and kinesin-driven motility [239]. Cdk5 positively controls TRPV1 membrane transport mediating KIF13B-TRPV1 association, without altering the total amount of TRPV1 [240]. Cdk5-dependent phosphorylation of KIF13B at Thr-506 is in part necessary for Cdk5-mediated motor-cargo association and contributes to efficient transport of TRPV1. Active Cdk5 promotes TRPV1 anterograde transport *in vivo* after inflammation [240], and regulates heat sensitivity and decreases of capsaicin-evoked calcium-influx in primary sensory neurons [241].

Finally, a new association of TRPV1 with Kvβ2 has been confirmed by co-immunoprecipitation assay in heterologous recombinant system in HEK as well as mouse DRGs. Kvβ2 exert chaperone-like effect resulting in an increased cell surface expression of TRPV1 associated with an enhancement in capsaicin sensitivity. These results suggest that this interaction may play a role in TRPV1 trafficking to the plasma membrane and could provide a structural basis of a spatially restricted repolarization pathway [231].

All of these reported protein-protein associations and signalling pathways evidence an enhanced exocytosis of TRPV1 to the plasma membrane; however, it is also possible to keep an increased surface expression of the channel by inhibiting its internalization. Persistent exposure to TRPV1 receptor agonists, such as the vanilloid capsaicin, makes cells partially or totally insensible to subsequent stimuli, a process known as desensitization. TRPV1 activation by capsaicin, temporary makes the channel unable to respond to the vanilloid or other agonists. Acute TRPV1 desensitization by capsaicin is a process completely dependent on Ca^2+^ and specific intracellular signalling cascades, mainly phosphorylation/dephosphorylation processes [242]. In contrast, capsaicin induces long-term receptor down-regulation by modulating the expression level of the channels promoting receptor endocytosis and degradation [243]. This process seems to be modulated by PKA-phosphorylation and it is mediated by clathrin- and dynamin- independent endocytosic mechanism.

Similarly, the E3-ubiquitin ligase Myc-binding protein-2 (MYCBP2, also known as PAM) regulates TRPV1 internalization through inhibition of P38MAPK signalling [244]. MYCBP2 is mainly expressed in peripheral and central nervous system, and it negatively controls neuronal growth and synaptic transmission [245]. Although loss of MYCBP2 seems not to cause fundamental changes in basal functions of sensory neurons, secondary inflammatory thermal hyperalgesia is significantly lower in MYCBP2 deficient mice [244]. There is an altered receptor trafficking in MYCBP2 deficient DRGs, and consecutive capsaicin-induced desensitization is not present in these nociceptors due to a decrease in TRPV1 internalization. Mechanistically, loss of MYCBP2 causes constitutive p38 MAPK activation that prevents activity-induced retrival of TRPV1 from the plasma membrane [246].

The above described protein-protein interactions mainly, but not exclusively, contribute to the constitutive trafficking of TRPV1. However, TRPV1 association with other proteins is involved in vesicle exocytosis upon stimulation by rapidly increasing the levels of this thermoTRP channel in the plasma membrane. In fact, this mechanism provides the basis for a rapid modulation of nociceptive TRPV1 responses and fine tuning of channel activity.

One of the first evidence was the finding that the N-terminus of TRPV1 associated with two well-known vesicular proteins, Snapin and Synaptotagmin IX, which bind to SNARE proteins and participate in neuronal regulated exocytosis [247]. Co-distribution of TRPV1 with Synaptotagmin IX and the vesicular protein synaptobrevin evidences TRPV1 located in vesicles near to the membrane surface. In this regard, PKC activation was able to induce a rapid delivery of functional TRPV1 channels to the plasma membrane from these vesicles, and the botulinum neurotoxin A (BoNT/A) blocked PKC-induced membrane translocation of this channel [247].

Subsequent findings demonstrate that TRPV1 membrane translocation can be induced by activation of several signalling cascades. For instance, activation of AC and PKA can lead also to transport of an intracellular pool of inactive TRPV1 monomers to the plasma membrane where they function as tetramers, without affecting total expression of the channel [248]. Another well-defined example is NGF that through activation of PI3K and Scr kinase, induces phosphorylation of TRPV1 and rapid insertion of new TRPV1 into the membrane surface [249]. This mechanism has been also shown for fibronectin-induced translocation of TRPV1 to the membrane of primary sensory neurons [250]. However, direct interaction of PI3K with TRPV1 can also promotes NGF-induced fast TRPV1 trafficking to the plasma membrane [251]. NGF is also able to promote long term up-regulation of TRPV1 translation and transport to the peripheral neuronal membrane through activation of P38MAKP pathway [252]. Interestingly, equivalent molecular mechanisms are shared by other stimuli such as insulin and insulin growth factor-1 (IGF-1) inducing enhancement of TRPV1 membrane trafficking through PI3K [253]. Based on these data, it is evident that activation of different signalling cascades promotes regulated exocytosis of a vesicle reservoir located near the plasma membrane. These vesicles contain new TRPV1 channels and they are transported to the membrane through SNARE-dependent mechanism highly sensitive to Ca^2+^. Consequently, it was found that TRPV1 membrane recruitment increased by several stimuli such as NGF, ATP or IGF-1 can be abolished by treatment with a botulinomimetic peptide, demonstrating how different signalling pathways convey only on regulated exocytosis [12].

In this line, the scaffolding protein A-kinase Ancoring Protein 79 (AKAP79, AKAP150 murine) allows the integration of several regulatory pathways. This anchor protein interacts with TRPV1 and has binding sites for PKA, PKC and calcineurin, forming a signalling complex that can regulate TRPV1 channel [254]. Overexpression of AKAP79/150 enhanced trafficking of TRPV1 to the membrane and conversely its down-regulation reduced TRPV1 membrane expression, although not completely [254]. Nevertheless, the main role of this association is on membrane trafficking of TRPV1 upon stimulation since the crossroad of several signalling cascades end up with TRPV1 translocation and phosphorylation of the channel. Interaction of AKAP79/150 has been also shown to other thermoTRP channels, especially TRPV4 but also TRPA1, TRPM8, TRPV2 or TRPV3.

Overall, it is important to note that there are two key features crucial for TRPV1 trafficking to the plasma membrane: one is phosphorylation and the other one is its mobilization through SNARE-dependent exocytosis.

#### Transport of TRPV1 in Neurons

TRPV1 is transported to the neuronal ends by different entities. The diffuse pattern of TRPV1 mostly matches well with the cytoplasmic and/or small vesicular distribution, but it can be also detected in structures with different and bigger size, mobility and recycling properties [255]. TRPV1 co-localizes with both pre- and post-synaptic proteins, and co-movements of TRPV1 with synaptic proteins, such as synaptophysin, can be observed. TRPV1 is detected in synaptic transport vesicles, and in transport packets within filopodia and neurites. Recycling and/or fusion of these vesicles can be rapidly modulated by TRPV1 activation, and as translocation of new TRPV1 channels to the plasma membrane of growth cones, can be measured immediately after addition of the endogenous TRPV1 agonist N-arachidonyl-dopamine [255,256]. This is mostly due to the translocation and subsequent fusion of readily available vesicles containing TRPV1. As a consequence it is also possible to define TRPV1 as a synaptic protein that regulates vesicle recycling [256,257].

It is well known that TRPV1 is expressed in peptidergic neurons that contain CGRP and SP. These neuropeptides are stored in LDCVs, and activation of TRPV1 induces release of both neuropeptides. Although still awaits demonstration, akin to other channels such as TRPV2 and DOR [258], it is reasonable to hypothesize that in peptidergic sensory neurons TRPV1 could be at least partially sorted into LDCVs. Therefore, as described for cortical neurons with synaptic vesicles [256], activation of TRPV1 not only would promote neuropeptides release but it also increase TRPV1 plasma membrane translocation. The enhancement of TRPV1 trafficking through a regulated exocytosis mechanism could be also the result of exogenous stimuli able to promote vesicle docking and fusion facilitating rapid modulation of neuronal excitability.

### 3.2. TRPV2

The analysis of protein-protein interactions with TRPV2 has revealed few insights in the mechanisms contributing to trafficking of this thermoreceptor. The only reported evidence is the association of TRPV2 with the recombinase gene activator protein (RGA) that tightly controls plasma membrane translocation of the channel in mast cells [259]. RGA is an intracellular transmembrane protein that transiently interacts with TRPV2 during cellular glycosylation. Although overexpression of RGA promotes an increase of basal TRPV2 in plasma membrane [260], RGA does not accompany TRPV2 to the cell surface. RGA is localized to a vesicular subcompartment of ER/Golgi apparatus functioning as a chaperone like and just promoting membrane trafficking of TRPV2 [259]. RGA seems to function as targeting protein during TRPV2 maturation controlling channel surface levels rather than a functional accessory subunit. Therefore, RGA interaction with this thermoTRP is a native complex determinant in biosynthesis and early trafficking of this channel [259,260].

An increased response of TRPV2 to certain factors has been studied through a mechanism that involves regulated access of new channels to the plasma membrane [260,261]. An elevation of cAMP is sufficient to drive TRPV2 to the plasma membrane of mast cells, thus any stimuli able to increase this secondary messenger may regulate TRPV2 trafficking. One of the first reported stimuli was IGF-1, which is able to promote TRPV2 recruitment to the membrane from the intracellular pools [262]. However, this is contradictory with the recent findings from Moiseenkova-Bell group, in which TRPV2 is reported to primarily reside in intracellular membranes and its subcellular distribution is not sensitive to IGF-1 treatment [263].

Another example is the exogenous application of insulin or high concentration of glucose which also stimulates translocation of TRPV2 to the plasma membrane in pancreatic beta cells [261]. The rapid exocytotic response induced by insulin is accompanied by membrane insertion of TRPV2 in a PI3K-dependent manner [261]. Similarly, depolarization of beta cells also induces translocation of TRPV2 to the membrane when exposed to K^+^ leading to increased entry of Ca^2+^ followed by enhanced insulin secretion [264]. Through a similar pathway, characterization of TRPV2 function and regulation in macrophages has revealed that TRPV2 translocation to the plasma membrane can be also promoted by the chemotactic peptide formyl Met-Leu-Phe, which is a PI3K-dependent mechanism sensible to pertussis toxin [265].

Alike TRPV1, but with lower affinity, TRPV2 interaction with the scaffold protein AKAP79/150 has been also reported [254]. Although until now no further research has been done on the functional implication of this association, we could speculate a comparable regulation as for TRPV1 whereby activity as well as translocation of the receptor to the membrane was modulated by this anchor protein.

The few data described above report that TRPV2 can be inserted to the plasma membrane though regulated exocytosis from an intracellular vesicular reservoir. This suggests that part of TRPV2 is partially sorted in appropriate competent vesicles. Unfortunately, there is still not enough evidence supporting this assertion, but recently TRPV2 has been associated to large-dense core vesicles from spinal cords homogenates by LC_MS and immunostaining. Further research is required to understand better the mechanism behind TRPV2 regulated exocytosis [258].

### 3.3. TRPV4

Mutation in TRPV4 gene results in some genetic disorders raising the medical and clinical interest of this thermoTRP channel. It seems that the environment and the interaction of other factors modulate oligomeraziation, trafficking and degradation of TRPV4, modifying channel activity [131].

The expression of TRPV4 in the plasma membrane depends on different factors. A complete and appropriate sequence of TRPV4 is required for channel membrane insertion. The C-terminal domain allows oligomerization of the channel and it is important not only for its trafficking and surface insertion but also for functional properties like selectivity and gating. In more detail, TRPV4 oligomerization promotes sorting from the ER to the cell surface, and complete deletion or site-specific mutations prevent membrane translocation resulting in partial or total retention of TRPV4 in the ER [266]. Consistently, new evidence further support the critical role of C-terminus in TRPV4 protein folding and trafficking. TRPV4 channels lacking 838–857 residues remain misfolded and fail to reach the Golgi apparatus [267]. These mutated channels suffer complex glycosylation and maturation, being subjected to degradation through the ER-associated degradation dependent pathway. On the contrary, mutation of Asn-651 into Gln shows an increase in constitutive membrane trafficking of TRPV4 without affecting overall channel expression in the cell. Since Asn-651 is located in the consensus N-linked glycosylation motif between S5 and S6, these results suggest that glycosylation of TRPV4 on this residue could influence membrane trafficking of the channel [268].

Several protein-protein interactions can tightly control the level and insertion of TRPV4 into the cell surface. For instance, the reticulum associated protein OS-9 interacts with the N-terminal tail of TRPV4 and facilitates proper folding and tetramer formation. OS-9 preferably binds to monomers and immature variants of TRPV4 and appears to protect TRPV4 from precocious ubiquitination and associated degradation. OS-9 acts as an auxiliary protein for TRPV4 maturation and impedes the release of the channel from the endoplasmic reticulum reducing its plasma membrane levels [269].

Another example is the novel association between the water channel AQP2 and TRPV4 that seems critical for Ca^2+^ entry during hypotonicity in renal cortical collecting duct cells [270]. TRPV4 plasma membrane expression is low under isotonic conditions, whereas hypotonic stimulation increases TRPV4 only in AQP2 expressing cells. TRPV1 membrane increase is inhibited by colchicine, a microtubule disrupting agent, showing the involvement of trafficking process. In this regard, TRPV4 is intimately associated with actin in CHO cells [271] through the C-terminus [272]. The phosphorylation in Ser-824 is a control point for its plasma insertion via the regulation of proper subcellular localization, being required for TRPV4 activity and protein stability [273]. Other researchers have previously reported that the TRPV4 phosphorylation at Tyr-110 residue is important in regulating its abundance at the plasma membrane [274]. Finally, TRPV4 can be also functionally down-regulated by WNK4 kinase via a decrease in the cell surface expression without affecting total abundance of TRPV4 [275]. Although a direct interaction between TRPV4 and WNK kinase has been not detected, a site mutation on WNK4 can abrogate the inhibitory effect on TRPV4 function and trafficking. However, it is necessary further investigation into the exact mechanism.

As mentioned for TRPV1, endocytosis can also modulate membrane expression of TRPV4 channels. The atrophin-interacting protein 4 (AIP-4) facilitates ubiquitination of TRPV4 and renders the channel available for endocytosis [276]. This process reduces TRPV4 surface expression and the internalized proteins are degraded by lysosomes. Mono or multi-ubiquinated TRPV4 channels are located in a vesicle pool below the plasma membrane, and overexpression of AIP-4 results in a reduction of TRPV4 basal activity due to an increased presence of TRPV2 in vesicles. On the contrary, interaction of TRPV4 with PACSIN 3 isoform regulates endocytosis enhancing the ratio of plasma membrane-associated *versus* cytosolic TRPV4 membrane [277]. In general, PACSINs are proteins implicated in synaptic vesicular membrane trafficking and regulation of dynamin-mediated endocytotic processes [278]. Co-expression of PACSIN 3 is suggested to increase cell surface expression of TRPV4 modulating its subcellular localization. A similar shift is also observable when dynamin-mediated endocytotic process is blocked. The interaction of PACSIN 3 is through the N-terminus of TRPV4 and it is required the specific proline-rich domain upstream of the ankyrin repeats. In conclusion, PACSIN 3 acts as a TRPV4 auxiliary protein that affects subcellular localization and modulates TRPV4 function in a stimulus-specific manner [98].

There are almost no data on stimulus-induced trafficking of homomeric TRPV4 channels. Xin Ma *et al.* [279] was the first group to identify the vesicular trafficking of heteromeric TRPV4-C1 channels to plasma membrane due to depletion of intracellular Ca^2+^ stores in HEK and vascular endothelial cells. Depletion by thapsigargin or by physiological agonists, such as bradykinin and ATP, caused an enhanced membrane translocation of TRPV4-C1 due to vesicular trafficking which was abolished by brefeldin A, but had little or no effect on TRPV4 or TRPC1 homomeric channels. One speculation could be that TRPV4-C1 heteromers are preferentially packaged into vesicles, thus their trafficking is more subjected to regulation by Ca^2+^ store depletion. In this regard, recent evidence indicates that the functional status of homomeric TRPV4 channels in the distal nephron is regulated by two distinct signalling pathways [110]: phosphorylation through PKC-dependent pathway increases the TRPV4 activity, while activation of the PKA-dependent cascade additionally promotes trafficking and translocation of TRPV4 to the apical membrane.

### 3.4. TRPM3

TRPM3 is a recently studied thermoTRP channel, thus until now there are only limited studies reporting some insights into the features and/or regulation of TRPM3 trafficking to the plasma membrane. Loss of 18 aa residues encoded by exon 13 partially diminishes channel insertion in the membrane [280]. This region, named the indispensable channel function (ICF), is required for TRPM3 channel function. Splicing variants within exon 13, such as TRPM3α7, are common in a variety of cell types and tissues. In fact, transcripts lacking ICF, are detectable in all TRPM3 expressing tissues, such as brain or DRG. These isoforms seem to interact, in a small degree, with other isoforms of TRPM3, possibly forming tetramers, but avoiding plasma membrane insertion. Apparently, ICF stabilizes interaction of TRPM3 subunits, essential for protein folding and allows cell surface insertion. It is interesting to mention that ICF region is also present in other members of the TRPM family such as TRPM8. Indeed, deletion of ICF also decreases expression of TRPM8 in cell surface, but in contrast the remaining channels in the plasma membrane are active.

### 3.5. TRPM8

Little is known so far about membrane trafficking of TRPM8. The only proposed mechanism reported that seem to regulate the transport and stabilization of TRPM8 to the plasma membrane are glycosylation and tetramerization [148].

Structurally, TRPM8 channels assemble as multimers using the putative coiled-coil region located in the C-terminus. Single-point mutation in this region, Leu-1089 into Pro, disrupts this interaction and reduces oligomerization and surface expression of TRPM8, without affecting total protein. Interestingly, although some monomers are able to reach plasma membrane, they are not able to form functional channels [148]. Within the N-terminal domain, two distinct regions have been recently identified, and they differentially contribute to channel activity and proper folding and assembly [281]. Deletion of region encompassing positions 40 to 60 aa is a key element in the proper folding and assembly of TRPM8, and augments responses to cold and menthol. In contrast, different deletions and site-directed mutations within this region rendered channels with an impaired function that are retained within the endoplasmic reticulum. Therefore, the initial region of the N-terminus is critical for the proper biogenesis of this thermoTRP channel.

N-glycosylation of TRPM8 takes place in Asn-934 in the extracellular loop between S5 and S6 near the pore region. First studies, reported that mutation of this residue into a glutamine reduced TRPM8 levels at the cell surface together with a concomitant reduction in channel activity [147,148]. These data also suggested that glycosylation was not an essential mechanism for TRPM8 to exit from the Golgi, since some channels still reach the plasma membrane. However, the impact of glycosylation on TRPM8 trafficking seems not so clear, since more recent reports have not been able to show any significant differences in the surface expression of this unglycosylated TRPM8 channels, although consistently temperature and menthol sensing is reduced [282,283].

Another proposed mechanism that could affect trafficking of TRPM8 is palmitoylation. Protein palmitoylation is a common post-traslational lipid modification which plays an important role in protein trafficking. TRPM8 has been identified to be efficiently palmitoylated by DHHC3 [284], although further research is needed to know whether palmitoylation plays some role and, if so, how it could affect membrane trafficking of the channel.

### 3.6. TRPA1

Many receptors and ion channels cycle between the plasma membrane and intracellular compartments, and the balance between membrane insertion and retrieval determines their surface abundance, and their activity [285,286]. Little is known about the TRPA1 trafficking, only Schmidt *et al.* [287] showed that the increased TRPA1 membrane availability observed upon MO application is at least partially dependent on SNARE-mediated vesicle-fusion and the other part might be via constitutive pathway. Only one work does reference to TRPA1 channel in the ubiquitination pathway showing that TRPA1 is a novel substrate for the de-ubiquitinating activity of the CYLD enzyme, and this de-ubiquitination causes a net increase in the cellular pool of TRPA1 proteins.

It is now believed that inflammation-mediated facilitation of trafficking of TRPV1 receptor channels plays an important role in the development and maintenance of inflammatory hyperalgesia. At the molecular level, it has been shown that TRPV1 surface expression could be regulated, in part, by SNARE-dependent exocytosis [12,247]. Wang *et al.* [206,217] reported that PKA and PLC signalling pathways sensitize MO-induced TRPA1 currents *in vitro*. Along this line, Schmidt *et al.* [288] showed that application of an activator of adenylyl cyclase and other of PLC-signalling, capsaicin, as well as activation of TRPA1 by MO significantly increased the levels of TRPA1 at the membrane of HEK cells and sensory neurons. The application of MO to DRG neurons induced an increase of the membrane capacitance which is indicative of the incorporation of new membrane channel into the neuronal surface. Tetanus toxin application selectively attenuated the response of cultured DRG to MO suggesting that the increased TRPA1 membrane availability observed upon MO application is at least partially dependent on SNARE-mediated vesicle-fusion. Recently, Burstein *et al.* [289,290] have proposed that, in addition to TRPV1, BoNT/A regulates SNARE-dependent cell-surface expression of TRPA1 channel.

As for TRPV1, the fact that TRPA1 trafficking to the plasma membrane can be induced by regulated exocytosis indirectly suggest the presence of this channel in secretory vesicles. In this regard, TRPA1 has been suggested to form a protein complex with secretogranin III [291]. Although the role of secretogranin III is poorly understood, it has been implicated in the biogenesis of secretory granules and this could indicate that TRPA1 might be stored in secretory vesicles.

## 4. A New Therapeutic Approach Targeting ThermoTRP Membrane Trafficking

The surface expression of ThermoTRP channels is controlled by constitutive and regulated vesicular trafficking. They are important mechanisms involved in assembly and trafficking of the channels to the plasma membrane and impact their function and regulation [285]. In fact, modulation of TRPV1 receptor surface density during pathological processes is nowadays considered as an interesting therapeutic approach for management of chronic pain, since an increase in trafficking is associated with the pathological state and does not seem to play a role in the physiological function of the channel [12,247].

Therefore, therapeutic strategies able to disrupt regulated SNARE-dependent exocytosis may have potential to treat pathologies with an associated increase in receptor membrane trafficking and vesicle exocytosis. The most evident and successful example is the use of botulinum neurotoxin (BoNT/A), known to disrupt SNARE complex formation by cleavage of the peripheral SNAP-25 protein, suppressing exocytosis of neurotransmitters, neuropeptides or receptors sorted in the vesicles [292,293]. BoNT/A modulates TRPV1 mobilization from the intracellular stores through regulated exocytosis [247,294]. Consistent with this, BoNT/A has been recently used to treat pain [295,296], showing beneficial effects in migraine [297,298], different neuropathic pain states [299,300], joint pain [301,302,303] and back pain [304]. This toxin reduces TRPV1 total expression inhibiting TRPV1 plasma membrane trafficking and renders TRPV1 vulnerable to ubiquitination and subsequent proteosomal degradation [289]. To improve the beneficial effects of BoNT/A, in the laboratory, protein engineering strategy has afforded several advances obtaining a potent, long-lasting and versatile inhibitor of exocytosis, a chimera toxin containing the protease of serotype E attached to the binding domain of serotype A with enhanced properties compared to native proteins, which could improve the treatment of chronic pain [305]. In this line, molecules that target regulated exocytosis and mimic botulinum neurotoxin effects offer an attractive therapeutic potential. Indeed, DD04107 is a palmitoylated peptide patterned after the N-terminus of SNAP-25 protein and is able to disrupt protein-protein interactions necessary to form SNARE-complex inhibiting regulated exocytosis [306]. This peptide is able to reduce inflammatory potentiation of TRPV1 in sensory neurons reducing TRPV1 membrane rapid translocation [12], and has demonstrated successful analgesic effect in animal models of inflammatory, neuropathic and bone-cancer induced pain [307].

## 5. Outlook

Modulation of the cellular expression of thermoTRP channels represents a fundamental cellular mechanism involved in the pathophysiology of these ion channels. The contribution of regulated exocytosis of these ion channels has been well documented for acute inflammatory sensitization of sensory neurons, although it remains yet elusive the underlying molecular details involved in the trafficking of these receptors to the cell membrane. Clearly, constitutive and regulated exocytotic routes coexist, at least in excitable cells, and appear to play in a concerted way to preserve the homeostasis of channel expression and to ensure fast recruitment of channels in response to an injury. However, the precise molecular components of both routes, as well as the type of vesicles used for the trafficking remain to be deciphered. Likewise, the contribution of the cellular context, which plays a key role defining the molecular composition of thermoTRP transport packets, remains largerly unknown. Furthermore, since the level of surface expression is finely tuned by the balance of exocytosis and endocytosis, understanding the molecular mechanism mediating receptor endocytosis appears also essential. Taken together, we have significantly progressed in this exciting field, although several questions are still requiring answer to fully understand the pathophysiological modulation of these ion channels and to identify novel targets for drug intervention that control their dysfunction by regulating the level of surface expression. A bright future in this research field is anticipated as the number of protein components potentially contributing to thermoTRP channel trafficking is still under intense scrutiny. The combination of complementary approaches, including *in vivo* life-imaging and systems biology will undoubtedly provide a comprehensive dynamical blueprint for the cellular trafficking of thermoTRPs and their modulation under different environmental conditions.
